# The relationship between anticholinergic drug burden and clinical frailty in urinary incontinence patients

**DOI:** 10.1590/1806-9282.20250075

**Published:** 2025-08-08

**Authors:** Baki Derhem, Mirac Ataman, Ercan Yuvanc

**Affiliations:** 1 Kırıkkale University, Faculty of Medicine, Department of Family Medicine – Kırıkkale, Turkey.; 2 Kırıkkale University, Faculty of Medicine, Department of Urology– Kırıkkale, Turkey.

**Keywords:** Cholinergic antagonists, Elderly, Frailty, Primary care, Urinary incontinence

## Abstract

**OBJECTIVE:**

The aim of this study was to investigate the relationship between anticholinergic drug burden and clinical frailty in patients with urinary incontinence.

**METHODS:**

This prospective cross-sectional study included 197 patients who were aged ≥60 years old and admitted to Family Medicine and Urology Departments of Kırıkkale Faculty of Medicine between May 2024 and August 2024. Anticholinergic burden was calculated using anticholinergic burden calculator, frailty was assessed using the clinical frailty scale, and these patients were assigned a frailty score ranging from 1 (very fit) to 9 (terminally ill).

**RESULTS:**

The mean age of the 197 participants in our study was 67.71±6.85 and 92 (46.7%) were female. The mean anticholinergic burden score was 2.43±1.77 and clinical frailty scale score was 4.75±1.05. There was a statistically significant difference between age groups, gender, marital status, educational status, anticholinergic burden status, and clinical frailty scale score (p<0.001, p=0.001, p=0.033, p<0.001, and p<0.001, respectively). There was also a statistically significant distance between age groups, gender, marital status, educational status, and anticholinergic aurden score (p=0.001, p=0.023, p=0.013, p=0.042, respectively). A strong, positive, and significant correlation was found between anticholinergic burden and frailty (r=0.728, p<0.01).

**CONCLUSION:**

The findings of this study highlighted that it is useful to evaluate the anticholinergic burden and reconsider treatment options when prescribing to elderly patients with urinary incontinence.

## INTRODUCTION

Urinary incontinence (UI) is a common condition in general population and, in particular, the older adult population, which reduces the quality of life of these people. Based on a meta-analysis, the prevalence of urinary incontinence in older adult women in the world is 37.1%^1^. The prevalence of UI in men over 65 years ranged from 11 to 34%^2^. Primary care clinicians are often in the best position to screen for, diagnose, and initiate treatment of UI. However, primary care clinicians are not routinely asking patients about this problem, either because of a lack of knowledge and confidence in UI treatment or because of the serious time limitations of routine primary care visits.

Antimuscarinic drugs act by blocking muscarinic receptor stimulation by acetylcholine and inhibiting smooth muscle contraction of the bladder. This blockade during storage leads to increased bladder capacity and decreased urgency. The ACB is defined as the cumulative effect of concomitantly taking multiple drugs with anticholinergic properties. It estimates the risk of suffering anticholinergic adverse effects. The ACB effect has a wide spectrum of side effects such as dizziness, blurred vision, urinary retention, constipation, confusion, and delirium. Higher ACB increases the hospitalization risk, functional loss, cognitive decline, morbidity, and mortality^3,4^.

In acute as well as chronic diseases, a well-known predictor for adverse outcomes is the loss of functional reserve, named frailty—a syndrome of reduced reserve and resistance to stressors and a multifactorial construct of a cumulative decline in different physiological systems. The measurement of frailty allows clinicians to help estimate the potential harms and benefits of intervention or treatment to patients^5^.

Symptoms, including declines in physical functioning, decreased cognitive functioning, and loss of appetite, can also occur independently with aging, while drugs with anticholinergic properties also have side effect profiles that contribute to these declines. Failure to recognize the drug-induced nature of these signs and symptoms may subsequently contribute to increased risk of frailty and subsequently increased risk of adverse events such as falls and fractures.

The negative effects of polypharmacy and multimorbidity on frailty, especially in elderly patients, have also been proven by various studies. The association of ACB with cognitive and functional impairments has been mostly investigated, but not many studies have examined the association between frailty and ACB.

The aim of this study was to investigate the effect of anticholinergic drug burden on clinical frailty in patients with UI.

## METHODS

This prospective cross-sectional study included 197 patients who were aged ≥60 years old and admitted to Family Medicine and Urology Departments of Kırıkkale Faculty of Medicine between May 2024 and August 2024. Ethical approval for the study was obtained from Kırıkkale Institutional Review Board (No: 2024.04.17).

Sociodemographic characteristics and medications used by patients diagnosed with UI were recorded by the investigators. Eligible patients were over 60 years of age, taking at least one prescription medication in regular treatment, and cognitively able to answer the questionnaires and give informed consent to the study. All medications used regularly (drugs being used for more than 1 month) for cardiovascular, neurological, urinary, or any other reason were recorded for ACB calculation. The antipsychotic drugs were not taken into consideration because of their well-known negative effects on cognitive function. Prescriptions of drugs with ophthalmic, otic, nasal, or topical routes of administration were not taken into consideration too. Patients who were ≤60 years old, didn't accept to participate in the study, and didn't meet the inclusion criteria were excluded also.

### The evaluation of anticholinergic burden and frailty

Many different scales have been developed to measure ACB, and their validity has been proven by studies on clinical outcomes. Expert rating scales are routinely used in research and clinical practice to quantify ACB. Expert opinion–derived rating scales generally rank the anticholinergic activity of drugs into four categories, ranging from no known anticholinergic activity (=0) to definite/high anticholinergic activity (=3). While there are multiple different scoring systems, the German Anticholinergic Burden score and the Anticholinergic Cognitive Burden Scale have been demonstrated to show the most validity and reliability^6^. Anticholinergic burden was calculated by using the anticholinergic burden (ACB) Calculator, which is a combination of these two scales (https://www.acbcalc.com/).

The measurement of frailty helps clinicians to estimate the potential harms and benefits of intervention or treatment to patients. Frailty was evaluated by using clinical frailty scale (CFS), which was developed by the Canadian Study of Health and Aging. The CFS is based on the clinical evaluation of a patient's status in the domains of multimorbidity, function, mobility, and cognition and has been validated in Turkish. The participants were assigned a frailty score ranging from 1 (very fit) to 9 (terminally ill). CFS evaluations were performed by one examiner who was trained in the use of CFS tool^7,8^.

### Statistical analyses

For all statistical analyses, IBM SPSS version 22 was used. Normality of variables was checked with Kolmogorov-Smirnov test. Mean±standard deviation was given for normally distributed values, while the median (minimum–maximum) was given for non-normally distributed values. Categorical variables were expressed with numbers and percentages. The Fisher's exact test and chi-square test were utilized for the comparison of categorical variables. For comparison of three groups’ continuous variables with non-normal distribution, Kruskal-Wallis H test was used. Then Mann-Whitney U test was performed on two groups with Bonferroni correction. A one-way ANOVA test was used for the comparison of normally distributed variables, and then post-hoc test with Tukey was performed for two groups’ comparisons. Relations between data were analyzed with Pearson or Spearman correlation analysis according to distribution. p<0.05 was considered significant for all tests.

## RESULTS

The mean age of the 197 participants in our study was 67.71±6.85 and 92 (46.7%) were female. While 25.4% of the respondents (n=50) were educated at high school and above, 51.8% (n=102) were educated at primary school and below. Notably, 184 (93.4%) of the respondents were not in any type of employment. While the smoking rate was 27.9%, the rate of alcohol consumption was found to be quite low (7.6%). Among all participants, 169 of all participants (85.8%) were married or living with a partner and 28 (14.2%) were living alone. The characteristics according to ACB score groups were given in Table 1.

**Table 1:** Basic characteristics according to anticholinergic burden score groups.

		ACB score=0 n=31	ACB score=1 or 2 n=77	ACB score ≥3 n=89	p-value
Age					
	60–64 years old	18 (22)	39 (47.6)	25 (30.5)	**0.006**
	65–74 years old	9 (11)	33 (36.6)	43 (52.4)	
	≥75 years old	4 (12.1)	8 (24.2)	21 (63.6)	
Gender n (%)					0.143
	Female	11 (12)	33 (35.9)	48 (52.2)	
	Male	20 (19)	44 (41.9)	41 (39)	
Smoking n (%)					0.177
	Active or ex-smoker	10 (10.9)	40 (43.5)	42 (45.7)	
	Never	21 (20)	37 (35.2)	47 (44.8)	
Marital status n (%)					0.078
	Married/with partner	29 (17.2)	69 (40.8)	71 (42)	
	Single/widowed	2 (7.1)	8 (28.6)	18 (64.3)	
Working status n (%)					0.696
	Employed	3 (23.1)	4 (30.8)	6 (46.2)	
	Not employed	28 (15.2)	73 (39.7)	83 (45.1)	
Educational status n (%)					0.112
	Primary school or below	12 (11.8)	38 (37.3)	52 (51)	
	Middle school	7 (15.6)	16 (35.6)	22 (48.9)	
	High school or above	12 (24)	23 (46)	15 (30)	

Note: Statistical differences were calculated as chi-squared test for categorical descriptors. ACB: anticholinergic burden. The bold values are considered statistically significant with p<0.05.

There was a statistically significant difference between age groups, gender, marital status, educational status, ACB status, and clinical frailty score (p<0.001, p=0.001, p=0.033, p<0.001, and p<0.001, respectively). The comparison of groups according to CFS score was given in Table 2.

**Table 2:** Comparison of demographics according to anticholinergic burden.

		Anticholinergic burden score (mean±SD)	p-value	p-value
Age (years old)				
	60–64^a^	1.90±1.61	**0.001**	**1–2=0.011**
	65–74^b^	2.67±1.70		**1–3=0.002**
	≥75^c^	3.12±1.81		**2–3=0.400**
Gender				
	Male	2.16±1.69	**0.023**	–
	Female	2.73±1.75		
Smoking status				
	Active or ex-smoker	2.48±1.59	0.697	–
	Never	2.38±1.87		
Marital status				
	Married/with partner	2.30±1.72	**0.013**	–
	Single/widowed	3.18±1.70		
Working status				
	Employed	2.15±1.51	0.561	–
	Not employed	2.45±1.76		
Educational status				
	Primary school or below^a^	2.65±1.72	**0.042**	1–2=0.899
	Middle school^b^	2.51±1.81		**1–3=0.034**
	High school or above^c^	1.90±1.63		2–3=0.199

Note: ANOVA was used to assess differences in means, then post-hoc test with Tukey was performed for two groups’ comparisons. SD: standard deviation. The bold values are considered statistically significant with p<0.05.

a,b,c indicate the groups, and the results of the comparison between groups after the post-hoc test with Tukey are given at the end.

There was a statistically significant difference between age groups, gender, marital status, educational status, and ACB score (p=0.001, p=0.023, p=0.013, p=0.042, respectively). The comparison of groups according to ACB score is given in Table 3.

**Table 3:** Comparison of demographics according to clinical frailty scale score.

		CFS score (mean±SD)	p-value	p-value
Age (years old)				
	60–64^a^	4.39±1.01	**<0.001**	**1–2=0.009**
	65–74^b^	4.85±0.94		**1–3=<0.001**
	≥75^c^	5.39±1.08		**2–3=0.024**
Gender				
	Male	5.52±1.03	**0.001**	–
	Female	5.01±1.01		
Smoking status				
	Active or ex-smoker	4.72±1.01	0.673	–
	Never	4.78±1.09		
Marital status				
	Married/with partner	4.69±1.01	**0.033**	–
	Single/ widowed	5.14±1.17		
Working status				
	Employed	4.31±0.94	0.116	–
	Not employed	4.78±1.05		
Educational status				
	Primary school or below^a^	5.01±1.02	**<0.001**	1–2=0.113
	Middle school^b^	4.64±1.02		**1–3=<0.001**
	High school or above^c^	4.32±0.99		2–3=0.209
Anticholinergic burden status				
	ACB=0^a^	3.68±0.87	**<0.001**	**1–2=<0.001**
	ACB=1 or 2^b^	4.36±0.72		**1–3=<0.001**
	ACB≥3^c^	5.46±0.84		**2–3=<0.001**

Note: ANOVA was used to assess differences in means, then post-hoc test with Tukey was performed for two groups’ comparisons. CFS: clinical frailty scale; SD: standard deviation; ACB: anticholinergic burden. The bold values are considered statistically significant with p<0.05.

a,b,c indicate the groups, and the results of the comparison between groups after the post-hoc test with Tukey are given at the end.

The relationship between ACB score (mean=2.43±1.77) and CFS score (mean= 4.75±1.05) was assessed by using Pearson correlation. A strong, positive, and significant correlation was found between these variables (r=0.728, p<0.01). There was also a significant but mild correlation between age and anticholinergic burden score (r=0.244, p=0.001), and there was no correlation between body mass index and ACB score (r=0.045, p=0.53).

## DISCUSSION

To our best knowledge, this is the first study to evaluate the correlation between frailty and ACB in patients with UI. Consistent with our hypothesis, in this study, we found a high positive correlation between ACB and frailty. We have shown that both frailty and ACB increase with age. We demonstrated that female gender, low educational level, and living alone negatively affected both CFS and anticholinergic burden.

In a systematic review published by Mehdizadeh et al. ACB drugs have been shown to be associated with frailty and reduced physical function in elderly patients, as well as increased risk of falls and mortality, but did not change the frailty status. In almost all of the articles included in this systematic review, frailty was classified as no frailty, pre-frailty, frailty, or severe frailty^9^.

Similar to our study, in a retrospective cohort study, a total of 1,453 adults aged 20–102 years were evaluated by Sargent et al., who found a significant association between ACB and cognitive decline (p=0.02), frailty (p<0.001), and cognitive frailty (p<0.001)^10^.

In another study of 824 HIV patients aged over 35 years, frailty was classified into two groups as frail and non-frail, and a strong relationship was reported between anticholinergic load and frailty (OR 1.32, 95%CI 1.05–1.66)^11^.

The analysis of longitudinal observational data from the ASPirin in Reducing Events in the Elderly (ASPREE) study stated that participants with higher ACB scores were older, had higher CESD-10 depression scores, were more likely to be minorities, female, and have fewer years of education, and were frail or pre-frail^12^. Similar to these findings, in our study, we also showed that ACB increased with female gender and low educational level. The earlier onset of aging in women due to hypoestrogenism may be interpreted as a factor here^13^.

A population-based cohort study of 2,087 participants aged 65 years or over at baseline (1992) living in South Australia, among the participants who were users of medicines with anticholinergic properties only, nonusers, users of medicines with sedative properties only, or medicines with sedative and anticholinergic properties, the participants who used anticholinergics or sedatives had three times or more the odds of being frail compared with nonusers (anticholinergics only: OR 3.9, 95%CI 2.9 to 5.3; sedatives only: OR 3.3, 95%CI 2.3 to 4.8; sedatives and anticholinergics: OR 6.2, 95%CI 4.6 to 8.5)^14^.

With 115 geriatric inpatients, Shwe et al. reported that only 44 patients (38.3%) were not exposed to any anticholinergic medication. Nearly two-thirds of patients were taking anticholinergic medications, with 25% classified as having a high anticholinergic burden (ACB≥4). Approximately one-third of severely frail patients were exposed to a high ACB. Patients with a high ACB were more than twice as likely to have severe frailty (OR 2.21; 95% confidence interval 1.05–4.6)^15^.

In a study conducted by Naharci et al., geriatric outpatients aged 65 years and older were classified as "pre-frail," "frail," and "robust" using the Fried frailty index, and anticholinergic load was divided into three groups such as ACB-0, ACB-1, and ACB-2 and over. In this study, they concluded that using drugs with possible or definite anticholinergic properties was associated with an increased risk of falls in frail older adults^16^. In our study, in line with the results here, we found that there was a statistically significant difference between the ACB-0, ACB-1,2, and ACB-3 groups when compared in terms of frailty (p<0.001).

In the cohort performed by Sargent et al., which included 80 individuals, they stated that five ACB tools evaluated in that cohort of low-income African American adults were highly correlated and predicted pre-frail and frail status^17^.

Both clinical frailty score and anticholinergic drug burden were significantly higher in the group aged 75 years and older. In a study conducted by Bagnoli et al. with 1,001 postmenopausal women, it was found that metabolic parameters deteriorated at the age of 75 years and older, especially systemic hypertension and decline in renal function. The fact that the drugs used in the control of these conditions can have anticholinergic properties is supportive of our findings^18^.

Although there is an increased risk of sarcopenia and osteoporosis and related falls and fractures in women in the postmenopausal period, the frailty score of men was found to be higher in our study^19^.

In the study performed by Joshi et al., patients with a diagnosis of schizophrenia or schizoaffective disorder were recruited from the community at five US universities as part of the Consortium on the Genetics of Schizophrenia–2. They found a higher anticholinergic medication burden was associated with worse cognitive performance, with an average ACB score of 3.8^20^. The reason why our mean score of ACB (2.43±1.77) was lower may be due to the fact that their study was conducted in patients with schizophrenia and schizoaffective disorder. This is because most of the drugs used to treat these disorders have a high anticholinergic effect.

The main limitations of our study are the small sample size and the fact that it was conducted as a single center in a tertiary hospital. But the exclusion of participants with a diagnosis of psychiatric illness and the use of scoring systems in addition to grouping in the assessment of both frailty and ACB allowed us to perform correlation analyses. This is an important strength of our study. Besides, patients with UI, which is a common disease encountered especially by primary care physicians, make our study valuable.

## CONCLUSION

UI is a chronic disease that is frequently encountered and managed in primary care. Considering the side effects of anticholinergic drugs used in the treatment of UI and the direct effects of polypharmacy, frailty should be evaluated, especially in elderly patients. It is useful to evaluate the ACB and reconsider treatment options, particularly when ­prescribing to elderly patients.

## Data Availability

The datasets generated and/or analyzed during the current study are available from the corresponding author upon reasonable request.
